# Supplementary material for: Recurring Cystitis: How Can We Do Our Best to Help Patients Help Themselves?
Source: Antibiotics (Basel). 2022 Feb 18;11(2):269. doi: 10.3390/antibiotics11020269 (PMC8868402; doi:10.3390/antibiotics11020269)
Supplement: Supplementary file 1 [file antibiotics-11-00269-s001.zip › antibiotics-1553094-supplementary.pdf]

antibiotics

Article

Recurring Cystitis: How Can We Do Our Best to Help Patients Help Themselves?

Sarah Ben Hadj Messaoud, Elisa Demonchy and Véronique Mondain *

Infectious Diseases Department, Université Côte d'Azur, Centre Hospitalier Universitaire de Nice, France; sarah.benhadjm@gmail.com (S.B.H.M.); demonchy.e@chu-nice.fr (E.D.)
* Correspondence: mondain.v@chu-nice.fr

Supplementary Materials

INFORMATION ON RECURRING CYSTITIS

1. **Background**

How old were you when you had your first episode of cystitis?

How old were you when you started getting these episodes more than 4 times a year?

How many yearly episodes do you get now?

Which trigger factors have you identified? (circle the correct propositions)

Sexual intercourse Digestive disorders: diarrhoea constipation Stress

Certain sports Other No identified trigger factor

Have you ever had fever or pyelonephritis? How often?

2. **Your symptoms** (circle the correct propositions)

Sudden onset Urinary frequency: during the day during the night Burning sensation

Incontinence Blood in the urine Smelly or cloudy urine

Bladder or pelvic pain Other

3. **Aside from your cystitis episodes**:

Do you have problems with passing urine? Frequency Incontinence

Difficulty voiding Other

4. **A few questions regarding your medical history, lifestyle or risk factors:**

How many litres do you drink each day on average?

Do you drink a lot of tea or coffee?

Do you « hold on » when you want to urinate?

Do you urinate avoiding sitting down so as not to touch the toilet?

How many times a day do you wash your intimate area? What product do you use?

Do you wear very tight clothes? A string?

Do you have digestive problems?

Irritable bowel syndrome Food allergies Other

Do you wipe from front to back when you've had a bowel movement?

Do you have a balanced diet? A good quality diet?

Have you had children? How many?

Vaginal deliveries? Perineal complications?

Are you menopaused?

Do you have vaginal dryness? Dyspareunia (pain during sexual intercourse)?

Do you take hormones? (contraceptive pill, hormone replacement therapy, oestrogen cream)

Do you know whether you have bladder or rectal prolapse (cystocele, rectocele)?

Do you regularly see a gynaecologist? When was your last gynaecological examination/pap smear

Are you a smoker? How many cigarettes a day? Since when?

Are there other women in your family who suffer from recurrent cystitis?

Do you practice any sports?

Have you any health problems at the moment?

Which problems?

What your long-term treatment do you take?

Do you have any drug allergies?

Have you undergone radiotherapy or chemotherapy?

Have you had urological or gynaecological surgery?

Do you have any known urological or bladder abnormalities? Neuropathic bladder Post-micturition residue

Hyperactive bladder Interstitial cystitis Other

Are you an anxious person? Do you sleep well?

Have you ever been assaulted during your life?

<u>Your experience</u>

Do you have an idea of the origin of this recurring cystitis?

What do you know about the causes of cystitis?

How does cystitis impact your daily life most?

What is your greatest fear related to these recurring episodes of cystitis?

<u>Your management, your care pathway</u>

What do you do when you get cystitis?

A urine test and culture: never sometimes systematically

Take a course of antibiotics: sometimes always Before the urine test results After the urine test result

Which microbe is usually identified?

Which antibiotics have you taken over the past six months: name, treatment duration

Have you taken long-term antibiotic prophylaxis: name, treatment duration

Do you do a urine test after treatment?

Do you do systematic urine tests (when you have a check-up, for instance, without any particular symptoms)?

Have you had an abdomen-pelvis utrasound examination recently? (less than 2 years ago)? (please bring it)

With a post-micturition residue examination?

Have you consulted a urologist?

What examinations have you undergone? Clinical examination Cystoscopy urodynamic testing

Do you take non-antibiotic medications for your cystitis episodes?

Phytotherapy: cranberry preparations other probiotics mannose other “alternative medicine»
